# Comparative analyses of serum parameters between nontransgenic and transgenic male chickens expressing the 3D8 single-chain variable fragment gene, and mortality and growth characteristics

**DOI:** 10.5713/ab.25.0046

**Published:** 2025-06-04

**Authors:** Hyeon Yang, Bo Ram Lee, Sun A. Ock, Mi-Ryung Park, Poongyeon Lee, Yong Jin Jo, Min Gook Lee, Jae Yong Han, Sung June Byun

**Affiliations:** 1 Animal Biotechnology Division, National Institute of Animal Science, Rural Development Administration, Wanju, Korea; 2 Department of Agricultural Biotechnology and Research Institute of Agriculture and Life Sciences, College of Agriculture and Life Sciences, Seoul National University, Seoul, Korea; 3 Animal Genetic Resources Research Center, National Institute of Animal Science, Rural Development Administration, Hamyang, Korea; 4 Poultry Research Institute, National Institute of Animal Science, Rural Development Administration, Pyeongchang, Korea

**Keywords:** Biometric, Chickens, Growth, Mortality, Transgenic

## Abstract

**Objective:**

Transgenic (TG) animals offer significant potential for diverse applications but may pose risks if the impact of transgene expression on health and physiological parameters is not thoroughly assessed. This study aims to evaluate the effects of 3D8 scFv gene expression on male TG chickens, focusing on key biological markers, mortality, and growth.

**Methods:**

Serum samples were collected at 14 weeks of age from male TG and non-TG chickens for comprehensive analysis of serum biochemistry, sex hormones, and cytokine profiles. Mortality and growth were monitored over a 34-week period to assess long-term effects. Statistical comparisons were conducted between TG and non-TG groups to identify significant differences.

**Results:**

The results revealed that male TG chickens showed significantly lower serum levels of alanine aminotransferase, insulin-like growth factor-1, interferon-gamma, interleukin-4, and tumor necrosis factor-1 alpha compared to non-TG chickens (p<0.05). However, no significant differences in mortality or final body weight were observed between groups (p>0.05). These findings are consistent with previous results in female TG chickens, indicating that 3D8 gene expression does not adversely affect health or growth performance.

**Conclusion:**

3D8 scFv gene expression in male TG chickens does not adversely affect mortality, growth, or serum parameters, indicating that the transgene does not have detrimental effects on these critical metrics in male chickens. These findings support the safety and physiological stability of 3D8 gene expression in TG animals.

## INTRODUCTION

Transgenic (TG) animals are being developed at an exceptional rate, especially for applications in agriculture, medicine, food production, and environmental adaptation [1–3]. While TG animals offer substantial promise, addressing the effects of transgene on health and physiology is essential to mitigating potential risks.

Serum parameters, sex hormones, and cytokines are pivotal biomarkers that reflect the physiological and immunological status of animals, offering a quantitative approach to health evaluation [4]. Among these, glucose (GLU), a primary energy substrate, provides insights into metabolic activity, while total protein (TP) and albumin (ALB) levels serve as indicators of dietary protein utilization and hepatic function [5,6]. Cytokines, such as interleukins and interferons, modulate immune responses by regulating the proliferation, differentiation, and activation of immune cells, thereby playing integral roles in maintaining homeostasis and defense mechanisms [7]. In TG animal models, deviations in these biomarkers can indicate potential physiological disruptions caused by transgene expression. Consequently, comprehensive profiling of serum biochemistry and cytokine levels is essential for investigating the physiological effects of transgene expression in TG animals.

Previous studies on 3D8 scFv TG chickens demonstrated no significant differences in mortality, growth, or egg productivity in female TG chickens compared to wild-type (WT) chickens [8,9]. However, these studies focused exclusively on female TG chickens, leaving the physiological and developmental impacts on male TG chickens unexplored. Sex-based variation in immune and hormonal regulation highlights the need to examine male TG chickens to comprehensively evaluate transgene effects. To address this gap, the present study examines serum parameters, sex hormones, cytokines, mortality, and growth in male 3D8 scFv TG chickens and compared these results with findings from female counterparts.

## MATERIALS AND METHODS

### Animal care and use

Experimental chickens were produced via artificial insemination (AI) using semen from male heterotypic 3D8 scFv TG and female WT White Leghorn (WL) chicken. The male 3D8 scFv TG chickens were generated using recombinant lentivirus and surrogate eggshell incubation method [8]. All chickens were managed under standardized housing, feeding, and vaccination schedules in accordance with the institute’s guidelines for animal health management to ensure consistent health status across groups. All experiments followed approved protocols from the Institute Animal Care and Use Committee (IACUC) of the National Institute of Animal Science (NIAS-2020-419) in the Republic of Korea.

### Polymerase chain reaction analysis

Whole blood was collected from newly hatched chicks and used for sex determination and transgene detection via polymerase chain reaction (PCR). PCR was performed using a Phusion Blood Direct PCR System (Thermo Fisher Scientific) and a SimpliAmp PCR system (Applied Biosystems). The primers for sexing were as follows: 5’-AGA ATG AGA AAC TGT GCA AAA CAG-3’ (forward) and 5’-CTA TCA GAT CCA GAA TAT CTT CTG C-3’ (reverse). The primers for transgene cassette detection were as follows: 5’-CCT CTG CTA ACC ATG TTC ATG CCT TC-3’ (forward) and 5’-GCT AGT GAA TGT GTA TCC AGA AGC CTT-3’ (reverse). The PCR procedure for sexing were as follows: 98°C for 5 min; 40 cycles of 98°C for 1 s, 53°C for 10 s, and 72°C for 15 s; 72°C for 1 min. The PCR procedure for transgene cassette were as follows: 98°C for 5 min; followed by 40 cycles of 98°C for 1 s, 62°C for 5 s, 72°C for 20 s; and 72°C for 1 min.

### Study population

A total of 210 chicks were hatched via AI, with 110 males (52.4%) and 54 male chicks (49.1%) testing positive for the 3D8 scFv transgene, consistent with Mendelian inheritance. Thirty male non-TG and 30 male TG chicks were randomly assigned to 5 experimental groups (n = 5, each group with 6 chickens). The chicks were housed in group cages until 17 to 18 weeks of age, after which they were transferred to individual cages. Lighting was adjusted from continuous light at hatching to 16 hours per day, following standard protocols.

### Serum biochemistry

A male non-TG chicken died before serum collection. Thus, the serum of male non-TG (n=29) and TG (n=30) chickens was collected at 14 weeks of age and stored at −20°C until use. The serum was analyzed via biochemistry. The concentration of GLU, calcium (CA), phosphorus (PHOS), TP, ALB, globulin (GLOB), alanine aminotransferase (ALT), alkaline phosphatase (ALP), and gamma-glutamyltransferase (GGT) were determined using an automated clinical chemistry analyzer (Hitachi Automatic Analyzer 7180).

### Evaluation of serum sex hormones and cytokine levels

Serum concentrations of sex hormones and cytokines were determined using commercial chicken enzyme-linked immunosorbent assay (ELISA) kits. Serum concentration of estradiol (n = 29, 30 in non-TG and TG groups, respectively) and testosterone (n = 29, 30) were determined using ELISA kits (Cusabio). The cytokines were insulin-like growth factor-1 (IGF-1), interferon-gamma (IFN-γ), interleukin-1 beta (IL-1β), interleukin-4 (IL-4), interleukin-6 (IL-6), interleukin-8 (IL-8), and tumor necrosis factor-1 alpha (TNF-1α). The serum concentrations of the IGF-1 (n = 29, 30), IFN-γ (n = 21, 27), IL-1β (n = 28, 29), IL-4 (n = 21, 30), IL-6 (n = 24, 21), IL-8 (n = 29, 30), and TNF-1α (n = 16, 17) were determined using ELISA kits. The IGF-1 ELISA kit was purchased from LSBio, and the IL-4 kit was purchased from Genorise Scientific. The remaining kits were purchased from Abebio.

### Mortality and growth measurements

The mortality and growth of the male non-TG (n=5) and TG (n=5) chickens were monitored daily from 1 to 34 weeks of age. Weekly averages of body weights and mortality rates were recorded and used for statistical analysis.

### Statistical analysis

Statistical analysis was performed using GraphPad Prism statistical software (GraphPad Prism 5.03 software). Student’s t test was used to compare serum parameters and cytokine and sex hormone levels between male non-TG and TG chickens, and a p-value of less than 0.05 was considered to indicate statistical significance. A mixed model ANOVA was used to compare mortality rates and body weights between the experimental groups of male non-TG and TG chickens, and a p-value of less than 0.05 was used to indicate statistical significance.

## RESULTS

### Serum biochemistry

Significant differences in several serum biochemical parameters were observed between non-TG and TG chickens at 14 weeks of age (Table 1). Compared to non-TG chickens, TG chickens exhibited notably lower serum concentrations of PHOS, TP, ALB, GLOB, and ALT (p<0.0001), and significantly higher levels of GLU, ALP, and GGT (p<0.05). These changes may reflect altered protein metabolism and liver enzyme activity in TG chickens.

### Serum sex hormones and cytokines

**Figure 1:**
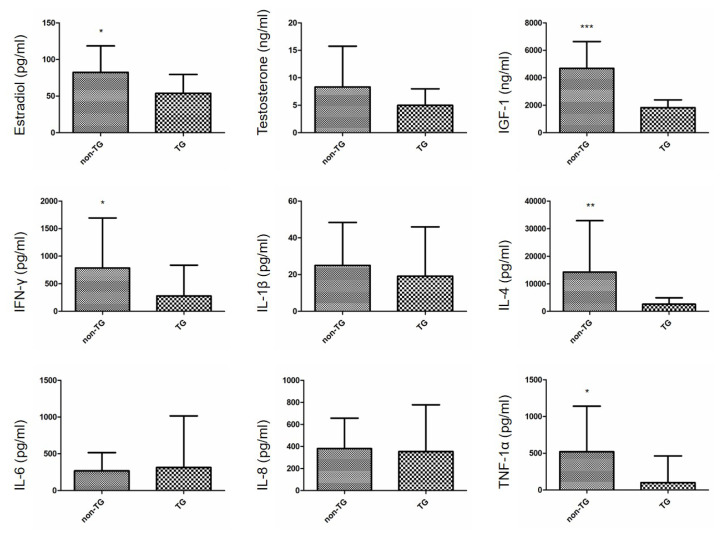
Serum sex hormone and cytokine levels in male non-TG and TG chickens at 14 weeks of age. ELISA analysis was performed to measure serum levels of estradiol (n = 29, 30 in the non-TG and TG groups, respectively), testosterone (n = 29, 30), IGF-1 (n = 29, 30), IFN-γ (n = 21, 27), IL-1β (n = 28, 29), IL-4 (n = 21, 30), IL-6 (n = 24, 21), IL-8 (n = 29, 30), and TNF-1α (n = 16, 17). Significant differences were observed in estradiol, IGF-1, IFN-γ, IL-4, and TNF-1α. Values are expressed as mean±standard deviation (SD). * p<0.05, ** p<0.01, *** p<0.001. IGF-1, insulin-like growth factor-1; IFN-γ, interferon-gamma; IL-1β, interleukin-1 beta; IL-4, interleukin-4; IL-6, interleukin-6; IL-8, interleukin-8; TNF-1α, tumor necrosis factor-1 alpha; TG, transgenic; ELISA, enzyme-linked immunosorbent assay.

Serum sex hormones and cytokines were observed between non-TG and TG chickens at 14 weeks of age (Figure 1). Compared to non-TG chickens, TG chickens showed significantly lower serum concentrations of estradiol (p<0.05), while testosterone levels remained comparable between the groups (p>0.05). Similarly, IGF-1, IFN-γ, IL-4, and TNF-1α levels were significantly lower in TG chickens (p<0.05), whereas IL-1β, IL-6, and IL-8 levels did not differ significantly (p>0.05). These results indicate that the expression of the 3D8 scFv gene may influence specific hormonal and immune parameters in male chickens. Although several cytokines showed significant differences between groups, the overall cytokine response pattern remained inconsistent, indicating a limited effect of transgene expression on immune regulation.

### Mortality and growth

**Figure 2:**
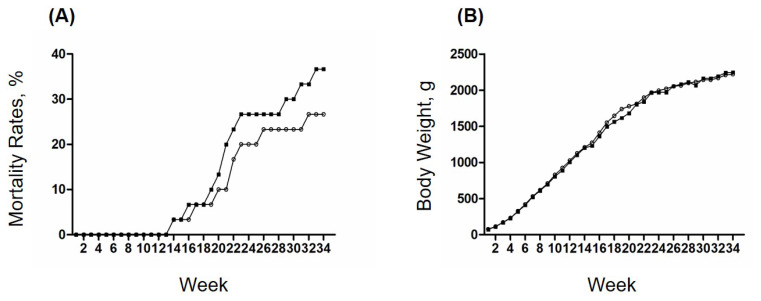
Mortality and growth in the experimental groups of male non-TG and TG chickens. The mortality rates and body weights of the male non-TG (n=5) and TG (n=5) chickens are shown. The weekly average values in the experimental groups of non-TG (empty circle) and TG (black square) chickens were used for analyses. (A) Cumulative mortality rates in the experimental groups of non-TG and TG chickens are displayed as percentages of the mean. (B) Body weights of the non-TG and TG chickens are displayed in grams of the mean. TG, transgenic.

Mortality and growth performances were measured between non-TG and TG chickens (Figure 2). Throughout the 34-week observation period, TG chickens showed a cumulative mortality rate of 36.7% (11 out of 30), compared to 26.6% (8 out of 30) in non-TG chickens; however, this difference was not statistically significant (p>0.05) (Figure 2A). Final body weight at 34 weeks was also similar between groups, with TG chickens averaging 2,246.5 g and non-TG chickens averaging 2,220.8 g (p>0.05) (Figure 2B). These findings suggest that the 3D8 scFv gene expression does not negatively impact survival or growth performance in male 3D8 TG chickens.

## DISCUSSION

The health and physiological status of experimental animals are commonly evaluated using diverse biochemical and immunological biomarkers. In this study, serum parameters such as ALB, GLOB, and the albumin-to-globulin (A/G) ratio were used to assess overall protein balance and liver function. A high A/G ratio can indicate potential pathological conditions, including liver, kidney, or intestinal disease [10]. Additionally, liver enzymes such as ALT and ALP serve as critical indicators of hepatic function [11].

The comparative analysis of serum parameters between male non-TG and TG chickens revealed significant differences (Table 1). Specifically, non-TG chickens exhibited higher levels of PHOS, TP, ALB, and GLOB compared to TG chickens. However, the A/G ratios were 0.64 and 0.59 for non-TG and TG chickens, respectively, suggesting that 3D8 scFv gene expression had minimal impact on overall protein distribution. Furthermore, serum biochemical parameters such as GLU, TP, and ALB were consistent with earlier studies [12,13]. These findings align with previous studies conducted on female non-TG and TG chickens [9], suggesting that differences in biochemical parameters may not meaningfully affect the physiological state of TG chickens.

Interestingly, ALT levels were significantly higher in non-TG chickens, whereas ALP levels were significantly elevated in male TG chickens (Table 1). Since ALT and ALP are liver-associated enzymes with distinct physiological roles, ALT being a marker of hepatocellular damage and ALP being associated with biliary or skeletal activity, the elevated ALP levels in TG chickens may suggest altered hepatic or skeletal function. However, no clinical symptoms were observed, indicating that the changes in enzyme levels may not reflect pathological significance. Previous studies comparing female non-TG and TG chickens also reported similar patterns [9]. However, neither this study nor previous research found definitive evidence that the expression of the 3D8 scFv gene directly affects liver or bone function. In addition to that, the mean ALT and ALP concentrations observed in this study (36.2 U/L and 189.4 U/L in non-TG; 17.7 U/L and 272.6 U/L in TG, respectively) differ from the reported baseline values for WT WL chickens [12]. This discrepancy suggests that further investigation is needed to clarify the role of the 3D8 scFv gene in ALP modulation.

In the present study, the mean concentrations of serum estradiol, IGF-1, IFN-γ, IL-4, and TNF-1α were significantly greater in non-TG chickens. These results showing significantly lower serum levels of cytokines in TG chickens were consistent with previous results obtained from female non-TG and TG chickens [9]. Although clear evidence that low levels of cytokines are directly driven by 3D8 scFv gene expression in animal model is limited, studies involving microbial expression of 3D8 scFv demonstrated cytokine reduction upon oral administration, suggesting a potential immunomodulatory effect of the gene product [14–16]. These findings indicate that further studies are needed to examine the direct impact of 3D8 scFv gene expression on cytokine regulation in TG animal models, including transcriptomic or proteomic analyses to identify gene expression patterns or pathways that may be modulated by 3D8 scFv expression.

Factors such as breed, age, diet, and vaccination schedules are known to influence serum parameters [17]. However, in this study, experimental conditions were standardized for both non-TG and TG groups, minimizing confounding variables. The differential levels of ALT, ALP, IGF-1, IFN-γ, IL-4, and TNF-1α between non-TG and TG chickens are, therefore, likely attributable to the expression of the 3D8 scFv gene. Importantly, consistent with previous studies in female TG chickens [9], these findings suggest that 3D8 scFv expression does not compromise the overall health or physiological outcomes of TG animals.

## CONCLUSION

In conclusion, this study demonstrates that 3D8 scFv gene expression alters specific serum parameters and cytokine levels in male TG chickens, while having no measurable impact on mortality or growth. The findings confirm the reproducibility of these effects across sexes and suggest that future studies should focus on identifying the molecular mechanisms responsible for these observations.

## Figures and Tables

**Table 1:** Serum parameters of male non-TG and TG chickens at 14 weeks of age

Parameters	Non-TG	TG	p-value
GLU (mg/dL)	233.21±12.61	243.30±19.07	0.0202
CA (mg/dL)	14.05±1.08	14.56±0.75	0.1017
PHOS (mg/dL)	6.54±0.72	3.81±0.76	<0.0001
TP (g/dL)	5.44±0.64	4.60±0.53	<0.0001
ALB (g/dL)	2.12±0.24	1.74±0.20	<0.0001
GLOB (g/dL)	3.32±0.44	2.86±0.37	<0.0001
ALT (U/L)	36.24±9.36	17.68±7.83	<0.0001
ALP (U/L)	189.38±41.71	272.63±89.07	<0.0001
GGT (U/L)	8.10±5.27	19.20±6.41	<0.0001

Serum collected from male non-TG (n=29) and TG (n=30) chickens at 14 weeks of age was used to analyze serum biochemical parameters, namely.

The data are presented as the mean±SDs.

TG, transgenic; GLU, glucose; CA, calcium; PHOS, phosphorus; TP, total protein; ALB, albumin; GLOB, globulin; ALT, alanine aminotransferase; ALP, alkaline phosphatase; GGT, gamma glutamyltransferase; SD, standard deviation.
